# Preliminary Study on Total Component Analysis and In Vitro Antitumor Activity of *Eucalyptus* Leaf Residues

**DOI:** 10.3390/molecules29020280

**Published:** 2024-01-05

**Authors:** Juanjuan Wu, Zixuan Wang, Xinying Cheng, Yunhe Lian, Xiaodong An, Di Wu

**Affiliations:** 1 Chenguang Biotech Group Co., Ltd., Handan 057250, China; 2 Chenguang Biotech Group HanDan Co., Ltd., Handan 056000, China; cxy@ccgb.com.cn; 3 Hebei Key Laboratory of Comprehensive Utilization of Plant Resources, Handan 057250, China

**Keywords:** *Eucalyptus* leaves, ultra performance liquid chromatography-quadrupole time of flight mass spectrometry, antitumor, composition analysis

## Abstract

*Eucalyptus globulus* is widely introduced and cultivated in Yunnan province. Its foliage is mainly used to extract eucalyptus oil, but the by-product eucalyptus residue has not been fully utilized. Based on the above reasons, in this study, we sought to explore the comprehensive utilization potential of eucalyptus resources. The total composition of eucalyptus residue was analyzed by ultra performance liquid chromatography-time-of-flight mass spectrometry (UPLC-Q/TOF MS), and the active components and nutrient components of eucalyptus leaf residue were determined by chemical methods and liquid phase techniques. Meanwhile, the antitumor activity of triterpenoids in eucalyptus leaves was evaluated by tetramethylazazole blue colorimetric assay (MTT). The results of qualitative analysis indicated that 55 compounds were identified from eucalyptus residue, including 28 phloroglucinols, 17 terpenoids, 3 flavonoids, 5 fatty acids, 1 amino acid and 2 polyphenols. Among them, the pentacyclic triterpenoids, in eucalyptus residue, were mainly oleanane type and urthane type. The results of quantitative determination indicated that the content of triterpenoid compounds was 2.84% in eucalyptus residue, which could be enhanced to 82% by silicone separation. The antitumor activity results showed that triterpenoid compounds have moderate inhibitory effects on human breast cancer cell MDA-MB-231, gastric adenocarcinoma cell SGC-7901 and cervical cancer cell Hela. The half maximal inhibitory concentration (IC_50_) was 50.67, 43.12 and 42.65 μg/mL, respectively. In this study, the triterpenoids from eucalyptus leaf residues were analyzed to reveal that the triterpenoids from eucalyptus leaf have antitumor effects and have potential to be developed as antitumor drugs.

## 1. Introduction

Eucalypt is the general name of the species of genus *Angophora*, *Corymbia*, and *Eucalyptus* of the Myrtaceae family, containing a total of 945 species and varieties that are important economic species naturally distributed in countries such as Australia [1]. Known for their fast growth, high yield, strong adaptability, and wide-ranging applications, eucalyptus trees have been introduced to China for over a century. Currently, more than 600 counties in over 20 provinces across China cultivate eucalyptus plantations [2]. Commonly planted eucalyptus species include *Eucalyptus globulus*, *Eucalyptus citriodora*, *Eucalyptus grandis*, and *Eucalyptus urophylla*, with major production regions in Guangdong, Guangxi, Yunnan, and Hainan [3].

One significant application of eucalyptus trees is for the production of eucalyptus essential oil. According to statistics, eucalyptus essential oil is a crucial export commodity for China, consistently ranking among the top four in export share from 2016 to 2020. *Eucalyptus* essential oil is well recognized for its anti-inflammatory, analgesic, anticancer, and antiviral properties, making it a valuable resource in the pharmaceutical industry [4]. Notably, *Eucalyptus globulus* cultivated in Yunnan has a high content of 1,8-cineole in its eucalyptus essential oil, exceeding 70%, making it a primary source for domestic eucalyptus essential oil production [5]. However, the residue after the extraction of eucalyptus essential oil, namely eucalyptus leaf residue, is not fully utilized. At present, eucalyptus residue is usually burned, which causes serious waste of resources and environmental pollution [6].

In this study, we focus on exploring the potential comprehensive utilization of eucalyptus leaf residue. Firstly, compounds, extracted from eucalyptus leaf residue with organic solvents of different polarity, were analyzed by ultra-high performance liquid chromatography-quadrupole time-of-flight mass spectrometry (UPLC-Q/TOF MS) and chemical methods. Subsequently, the extract was separated and purified by silica gel column chromatography, and high purity triterpenoids were obtained. Then, the efficacy of triterpenoids was evaluated through in vitro antitumor activity, aiming to provide insights into the pharmacological research of active components in eucalyptus leaf residue and offer a basis for the comprehensive utilization of eucalyptus leaf resources.

## 2. Results and Discussion

### 2.1. Qualitative Analysis of Components in Eucalyptus Leaf Residue

**Figure 1:**
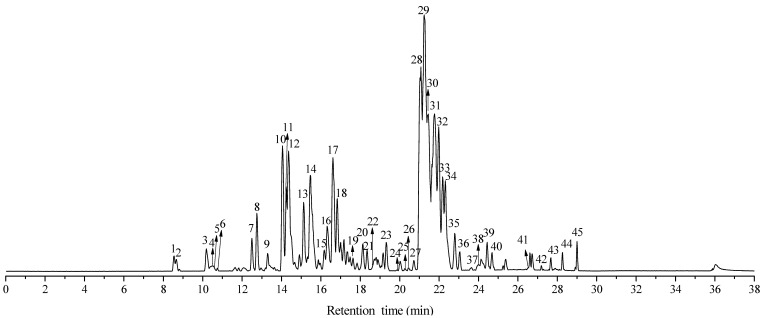
Total ion chromatography (TIC) of *n*-hexane extracts of *Eucalyptus* leaf residue in negative modes.

**Figure 2:**
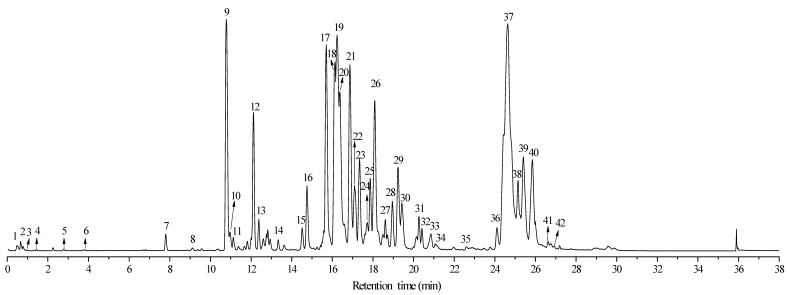
Total ion chromatography (TIC) of 70% ethanol extracts of *Eucalyptus* leaf residue in negative modes.

As described in Section 3.3, the chemical components in *n*-hexane and 70% ethanol extracts of eucalyptus leaf residue were separated and identified using UPLC-Q/TOF MS. Figure 1 and Figure 2 display the total ion chromatograms of *n*-hexane and 70% ethanol extracts of eucalyptus leaf residue in negative ion mode. From the figures, it can be observed that both extracts are primarily composed of phenolic compounds and triterpenoids, with similar compositions but varying in component concentrations. A total of 55 compounds were identified in this study, including 28 phloroglucinols, 17 terpenoids, 3 flavonoids, 5 fatty acids, 2 polyphenols, and 1 amino acid. The specific components are listed in Table 1 and Table 2.

#### 2.1.1. Triterpenoids

Triterpenoids are a class of molecules with the molecular formula (C_5_H_8_)_n_ that are derivatives of isoprene. Depending on the number of isoprene units in the molecular structure, they can be classified as monoterpenes (n = 2), sesquiterpenes (n = 3), diterpenes (n = 4), triterpenes (n = 6), tetraterpenes (n = 8), and polyterpenes (n > 8), and are widely distributed in nature. Most terpenoids in nature are oxygen-containing derivatives, typically alcohols, aldehydes, ketones, carboxylic acids, esters, and glycosides.

The compounds were identified through the analysis of secondary mass spectrometry fragment ion information, literature review, and the understanding of mass spectrometry fragmentation patterns. According to Table 1 and Table 2, a total of 17 terpenoids were identified in eucalyptus leaf residue, mainly comprising monoterpenes, sesquiterpenes, and triterpenes. Terpenoids in negative ion mode were primarily present in the form of [M − H]^−^. Among them, nine compounds belonging to monoterpenes and sesquiterpenes were identified, including eucalyptol, eucaglobulin, eucalmaidin D, and some formyl-substituted terpene components. Eight triterpenoids were identified, including five triterpenes mainly of the dammarane and ursane types, and three containing coumaryl substitutions [7].

**Figure 3:**
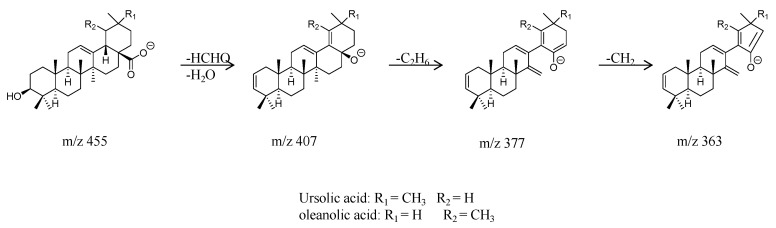
Fragmentation pathway of oleanolic acid or ursolic acid.

Analysis of pentacyclic triterpenoids: Taking compound peak 10 (Figure 1) as an example, its retention time was 13.46 min, and the quasi-molecular ion peak in negative ion mode was [M − H]^−^ at *m*/*z* 455. In the secondary mass spectrum, the sub-ion [M−H−HCHO−H_2_O]^−^ at *m*/*z* 407 was observed, indicating the loss of formaldehyde and water. Further removal of C_2_H_6_ formed the sub-ion peak [M−H−HCHO−H_2_O−C_2_H_6_]^−^ at *m*/*z* 377, and subsequent removal of CH_2_ resulted in the sub-ion peak [M−H−HCHO−H_2_O-C_2_H_6_−CH_2_]^−^ at *m*/*z* 363, with an additional ion peak at *m*/*z* 248 due to the Retro-Diels–Alder reaction. Based on mass spectrometric information and literature [8], it was inferred that compound **10** is dammarane/ursane/betulinic acid, and its fragmentation pattern is shown in Figure 3.

**Figure 4:**
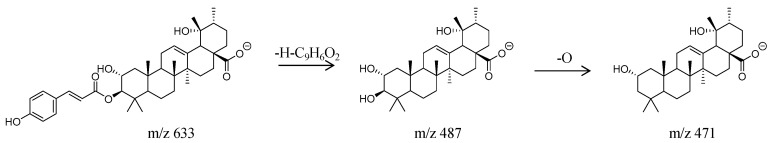
Fragmentation pathway of 3-O-*trans*-*p*-Coumaroyltormentic acid.

Analysis of coumaryl-substituted pentacyclic triterpenoids: Taking compound peak 37 (Figure 1) as an example, its retention time was 23.62 min, and the quasi-molecular ion peak in negative ion mode was [M − H]^−^ at *m*/*z* 633. In the secondary mass spectrum, a triterpene product ion peak [M−H−C_9_H_6_O_3_]^−^ at *m*/*z* 471 was observed, corresponding to the loss of O-*p*-coumaryl (162 Da), and an additional ion peak at *m*/*z* 248 due to the Retro-Diels–Alder reaction. Based on the above information and literature [9], it was inferred that peak 37 is 3-O-*trans*-*p*-Coumaroyltormentic acid, and its fragmentation pattern is shown in Figure 4.

#### 2.1.2. Phloroglucinols

Phloroglucinol and its derivatives are a unique class of compounds specific to *Eucalyptus* plants, characterized by distinctive structures. They often combine with monoterpenes, sesquiterpenes, and diterpenes to form novel compounds. Due to the diverse structures of the associated monoterpenes, sesquiterpenes, and diterpenes, a wide variety of phloroglucinol derivatives are formed. According to literature reports, phloroglucinol derivatives are mainly classified into three types of compounds: phloroglucinols, phloroglucinol dimers, and hybrids formed by phloroglucinols with monoterpenes, sesquiterpenes, or diterpenes. In eucalyptus leaf residue, a total of 28 phloroglucinol components were identified. Some are phloroglucinol dimers, primarily including Macrocarpal A and Sideroxylonal A/B/C [10,11,12]. Others are hybrids formed by phloroglucinols with monoterpenes, sesquiterpenes, or diterpenes, mainly including eucalyptone, eucalyptal, Macrocarpal A/B/D/E, Macrocarpal C, and Macrocarpal I/J [7,12,13].

**Figure 5:**
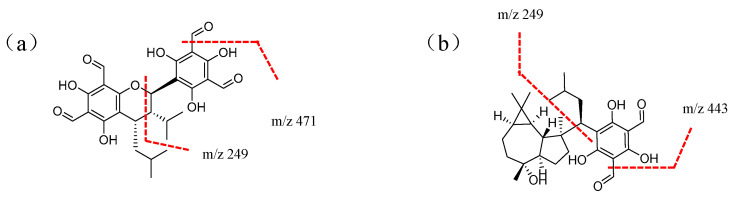
Fragmentation pathway of Sideroxylonal A (**a**) and Macrocarpal A (**b**).

Analysis of phloroglucinol dimers: Taking compound peak 13 (Figure 1) as an example, its retention time was 15.14 min, and the quasi-molecular ion peak in negative ion mode was [M − H]^−^ at *m*/*z* 499. In the secondary mass spectrum, the quasi-molecular ion peak [M−H−CO]^−^ at *m*/*z* 471, formed by the removal of CO, was observed. This ion further dehydrated to form the [M−H−CO−H_2_O]^−^ at *m*/*z* 453 sub-ion. Additionally, the base peak at *m*/*z* 249 of isopentyl dimethylphloroglucinol [C_13_H_13_O_5_]^−^ was observed. Based on the above information and literature [14], it was inferred that compound **13** is Sideroxylonal A/B/C, and its fragmentation pattern is shown in Figure 5a.

Analysis of hybrids formed by phloroglucinols with monoterpenes, sesquiterpenes, or diterpenes: Taking compound peak 11 (Figure 1) as an example, its retention time was 14.07 min, and the quasi-molecular ion peak in negative ion mode was [M − H]^−^ at *m*/*z* 471. In the secondary mass spectrum, base peaks [M−H−H_2_O]^−^ at *m*/*z* 453 and [M−H−CHO]^−^ at *m*/*z* 443, formed by dehydration and removal of CHO, respectively, were observed. Additionally, the base peak at *m*/*z* 249 of isopentyl dimethylphloroglucinol [C_13_H_13_O_5_]^−^ was detected. The fragmentation pathways observed were consistent with the mass spectrometric fragmentation reported for Macrocarpal A/B/D/E, suggesting that compound **11** is composed of Macrocarpal A/B/D/E, and its fragmentation pattern is shown in Figure 5b.

#### 2.1.3. Flavonoids

Flavonoids are widely present in plants in nature and belong to the secondary metabolites of plants. Their basic skeleton is C6-C3-C6, and depending on the different substituents, they can be classified as flavones, anthocyanins, chalcones, flavonols, and so on. Different types of flavonoid glycosides and aglycones exhibit characteristic fragmentation patterns. Flavonoid aglycones are prone to undergoing neutral losses (such as the loss of sugar, -CH_3_, -CO, -H_2_O, -CH_2_O, and other free radicals) or undergoing Retro-Diels–Alder (RDA) reactions at the glycoside position, producing characteristic fragment ions.

In eucalyptus leaf residue, three flavonoid compounds were identified, including Sideroxylin, Eucalmaidin D/cypellogin A/B, and leptospermone. The fragmentation patterns of these flavonoids are consistent with the literature-reported data for O-flavonoid glycosides.

**Figure 6:**
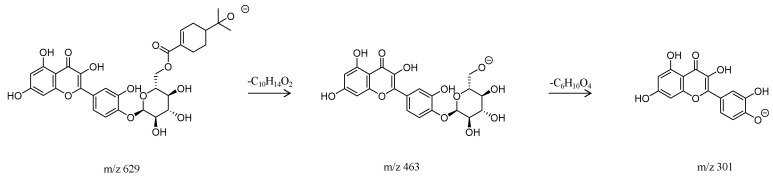
Fragmentation pathway of Eucalmaidin D.

Analysis of flavonoids: Taking compound **4** (Figure 2) as an example, its retention time was 1.43 min, and the quasi-molecular ion peak in negative ion mode was [M − H]^−^ at *m*/*z* 629. In the secondary mass spectrum, the base peak [M−H−C_10_H_14_O_2_]^−^ at *m*/*z* 463, formed by the loss of olivanic acid, was observed. This fragment ion further lost deoxyhexose to form the base peak [M−H−C_10_H_14_O_2_−C_6_H_10_O_4_]^−^ at *m*/*z* 301 (quercetin). Combining the fragmentation pattern of the compound with literature data [15,16], it was inferred that the compound was Eucalmaidin D/cypellogin A/B, and its fragmentation pattern is shown in Figure 6.

### 2.2. Quantitative Analysis of Components in Eucalyptus Leaf Residue

Based on the quantitative method in Section 3.4.4, the active components (total sugars, total terpenes, phloroglucinol derivatives) and nutritional components (crude fiber, crude protein, crude fat, crude ash, moisture) of eucalyptus leaf residue were determined, with a total content of 76.99%. The content of each component in the eucalyptus leaf residue is shown in Table 3.

The content of terpenoids in eucalyptus leaf residue accounted for 2.84% of the raw material. Studies displayed that pentacyclic triterpenes, found in *Eucalyptus*, have inhibitory effects on cancer cells in the esophagus, lung [17], liver [18], breast [19], pancreas [20], colon, and stomach. The anti-tumor mechanism was mainly achieved by blocking the cell cycle of tumor cells, regulating the protein expression of related genes, inhibiting cell proliferation, inducing cell differentiation, and regulating the body’s immune response. In the early stages of cancer treatment, patients might choose treatment drugs with minimal adverse effects or high efficiency at lower doses [21]. Therefore, the pentacyclic triterpenes, from eucalyptus leaves, are expected to be developed as candidate anti-tumor drugs.

The content of phloroglucinol derivatives in eucalyptus leaf residue accounted for 1.93% of the raw material. Research has shown that phloroglucinol derivatives have good biological activities such as antibacterial, antiviral, and anti-tumor effects, with broad prospects for medical research, and are expected to become new anti-tumor and antibacterial drugs [22].

*Eucalyptus* leaf residue contains a small amount of flavonoids. Studies indicated that flavonoids in eucalyptus leaves have antibacterial, anti-cardiovascular, anti-inflammatory, analgesic and significant antioxidant properties, which could be developed for new, natural antioxidant-containing functional foods and applied widely in the pharmaceutical field [23].

The content of crude fiber in eucalyptus leaf residue was 27.6%, and the content of crude protein was 5.64%. Due to the high content of crude fiber, it could be used as fiber feed (the content of crude fiber ≥ 18%) in the feed industry.

### 2.3. In Vitro Antitumor Activity

In order to evaluate the antitumor activity of triterpenoids (content of 82.55%) from eucalyptus leaf residue, fluorouracil (5-FU) was chosen as the positive control drug. MDA-MB-231, SGC-7901, and Hela tumor cells were selected as experimental subjects, and the triterpenoids were tested for activity using the MTT (4,5-dimethylthiazol-2-yl)-2,5-diphenyltetrazolium bromide) colorimetric method.

The results, as shown in Table 4, demonstrated a certain antitumor activity of triterpenoids from eucalyptus leaf residue. Compared to positive drug (5-FU), they demonstrated moderate inhibitory activity against MDA-MB-231, SGC-7901, and Hela cells, with half-maximal inhibitory concentration (IC_50_) values of 50.67, 43.12, and 42.65 μg/mL, respectively. Additionally, Wu et al. [24] studied the growth inhibition rate of mango saibao total triterpenes (TTC) on H_22_ liver cancer cells. Results showed tumor inhibition rates of 42.65% and 43.63% for the high-dose groups (200 mg/kg and 400 mg/kg), respectively. Liang et al. [25] investigated the effects of total triterpenes from *Celastrus orbiculatus* on the proliferation, apoptosis, and invasion of human esophageal cancer Eca-109 cells. Studies revealed an inhibitory effect, with a maximum inhibition rate of 44.69% at a drug concentration of 160 μg/mL, indicating a dose–response relationship. Ma et al. [26] explored the antitumor activity of different doses of triterpenoid compounds from *Laurencia*, and results showed an inhibition rate of 39.1% in the high-dose group (63.29%, 100 mg/kg).

Compared to positive drug (5-FU) and triterpenoid compounds from other plant sources, the antitumor activity of the triterpenoids from eucalyptus leaf residue was stronger than that of other plant triterpenes, and weaker than that of positive drugs (5-FU). To summarize, triterpenoids found in eucalyptus leaf residue exhibit moderate antitumor activity and hold potential for further development. The data from this study provided insights and theoretical support for pharmaceutical research on the effective components of eucalyptus leaves.

## 3. Experimental Section

### 3.1. Instruments and Apparatus

Ultra performance liquid chromatography-quadrupole time-of-flight mass spectrometry (UPLC-Q/TOF MS), BEH C18 column (2.1 × 100 mm, 1.7 μm): Waters corporation, Milford, MA, USA; DK-98-2 water bath: Tianjin test company, Tianjin, China; MM400 retsch grinder: Leica company, Wetzlar, Germany.

### 3.2. Materials and Reagents

*Eucalyptus* leaves were collected from Yunnan. The eucalyptus leaf residue was obtained after the extraction of essential oil from eucalyptus leaves by the method of steam distillation. The residue was dried, ground, sifted through a 40–60 mesh sieve, and stored for extraction.

Ethanol and *n*-hexane from Yongda Reagent (Tianjin, China); Acetonitrile from Fisher (Waltham, MA, USA); Deionized water (18.2 MΩ·cm) from Millipore Milli-Q plus (Haverhill, MA, USA) system. Solvents were of analytical grade for extraction purposes and LC/MS grade for UPLC-Q/TOF MS.

### 3.3. Sample Preparation and Enrichment

Preparation of Analysis Samples: Firstly, 10 g eucalyptus leaf residue was weighed and transferred to a conical flask, followed by the addition of 60 mL of *n*-hexane for extraction at 50 °C for 3 h. The extract was then filtered and concentrated to obtain an *n*-hexane concentrate with a yield of 4.38%. Subsequently, the remaining residue was further extracted with 60 mL of 70% ethanol at 80.0 °C for 3 h, after which the extract was filtered and concentrated to obtain a 70% ethanol concentrate with a yield of 10.56%. Then, the *n*-hexane and 70% ethanol extracts were redissolved, diluted to 3.0 mg/mL, and filtered through a 0.22 μm membrane into the liquid phase injection vial for UPLC-Q/TOF MS analysis.

Isolation of Triterpenoid Components: Firstly, 10 g eucalyptus leaf residue was weighed and transferred to a conical flask, followed by the addition of 60 mL of 70% ethanol for extraction at 85 °C for 3 h. The extract was then filtered and concentrated to obtain ethanol extract. Subsequently, the triterpenoids, with high purity (content > 80%), were obtained from ethanol extract by silica gel column chromatography using a mixture of *n*-hexane and ethyl acetate (*v*:*v*, 2:1) as eluent, and were used for in vitro antitumor experiments.

### 3.4. Experimental Methods

#### 3.4.1. Chromatographic Conditions

Chromatographic column: Waters Acquity UPLC BEH C18 (2.1 × 150 mm, 1.7 μm); Mobile phase: A was 0.1% formic acid in water, and B was acetonitrile or isopropanol: acetonitrile (*v*:*v*, 1:1); Elution conditions: 0–30 min (25–100%B), 30–35 min (100%B), 35–35.5 min (100–25%B), 35.5–38 min (25%B); Flow rate: 0.3 mL/min; Column temperature: 40 °C; Injection volume: 1.0 μL.

Due to the different polarities of *n*-hexane and 70% ethanol extracts, different elution solvents were used to ensure the complete elution of components. *n*-hexane extract was eluted and separated using 0.1% formic acid in water and isopropanol: acetonitrile (*v*:*v*, 1:1) as the mobile phase, and 70% ethanol extract was eluted and separated using 0.1% formic acid in water and acetonitrile as the mobile phase.

#### 3.4.2. Mass Spectrometry Conditions

Electrospray ionization source (ESI) in negative ion mode was used to collect MS^E^ data. Calibration solutions were 200 pg/μL leucine enkephalin solution and 0.5 mmol/L sodium formate solution. The scan range was *m*/*z* 50–1200, with a scan time of 0.5 s. In negative ion mode, the capillary voltage was 2.5 kV, cone voltage was 40 V, ion source temperature was 120 °C, and high-purity N_2_ was used as the auxiliary spray ionization and desolvation gas. The desolvation gas temperature was 400 °C, and the flow rate was 800 L/h [27,28].

#### 3.4.3. Data Analysis

Mass spectrometry data were collected and processed using Masslynx V4.1 software. The UNIFI scientific information system was used for data browsing, storage, and comprehensive analysis. Component identification was performed by extracting MS spectra and related MS/MS information, based on built-in mass spectrometry analysis platforms, including ChemSpider online databases (LIPID, Hmbd.ca, etc.) and traditional Chinese medicine databases (TCM Chinese [UNIFI 1.7]), combined with literature information [29,30,31].

#### 3.4.4. Quantitative Methods

Total sugar, total phenol, total triterpenoid, and moisture content in the extracts were determined using the phenol-sulfuric acid method, Folin-phenol method, vanillin-aluminum chloride method, and 105 °C oven method, respectively. Crude fiber, crude protein, crude fat, and crude ash content in eucalyptus leaf residue were determined according to [GB/T 5009.10-1985] [32], [GB/T 5009.5-2016] [33], Soxhlet extraction method, [GB/T 23742-2009] [34], and [GB/T 5009.124-2016] [35].

#### 3.4.5. Efficacy Experiments

##### In Vitro Antitumor Activity Experiments

Cancer cells (human breast cancer cells MDA-MB-231, human gastric adenocarcinoma cells SGC-7901, and human cervical cancer cells Hela) were cultured in DMEM medium at 37 °C and 5% CO_2_ until logarithmic growth phase. Cells in logarithmic growth phase were digested with trypsin, and 10 μL of cell suspension was transferred to a 96-well plate. After 24 h of adherent culture, 100 μL of different concentrations of triterpenoid solution (mass concentrations of 0, 6.25, 12.5, 25, 50, and 100 μg/mL) was added. After 48 h of incubation, 100 μL of CCK-8 reaction reagent (10:1) was added, mixed, and incubated for an additional 1.5 h at 37 °C and 5% CO_2_. The liquid in the wells was then removed, and 200 μL of 0.1% dimethyl sulfoxide (DMSO) was added to each well, followed by oscillation for 10 min to dissolve the crystals [36,37]. Absorbance values (OD values) were detected at 450 nm using a microplate reader. The negative control group was the DMSO, and each experimental group was set up in 5 replicate wells. The inhibition rate of triterpenoids on cancer cells was calculated using Formula (1):

(1) $$Inhibitionrate=(1-\frac{OD\left(Samplecontrol\right)}{OD\left(Negativecontrol\right)})\times 100\%$$

##### Statistical Analysis

GraphPad Prism 8 statistical software was used for statistical analysis, and Microsoft Excel 2010 was used to assist in calculating the half-maximal inhibitory concentration (IC_50_) values.

## 4. Conclusions

This study employed UPLC-Q/TOF MS to isolate and identify the chemical components in eucalyptus leaf residue. A total of 55 chemical components were preliminarily identified, including phloroglucinol derivatives, terpenoids, flavonoids, polyphenols, organic acids, amino acids, and 77% of the chemical components of eucalyptus leaf residue were revealed. Furthermore, the residue was abundant in phloroglucinols and triterpenoid compounds, with contents of 1.93% and 2.84%, respectively, making it a valuable natural source for these compounds. Additionally, the triterpenoid compounds in eucalyptus leaf residue exhibited moderate inhibitory effects on MDA-MB-231, SGC-7901, and HeLa cells, indicating their potential as promising candidates for anticancer drug development. This research offers a framework for pharmaceutical studies on the active components of eucalyptus leaves, thereby establishing a theoretical foundation for the rational development and utilization of eucalyptus leaf residue resources.

## Figures and Tables

**Table 1:** Compounds identified from *Eucalyptus* leaf *n*-hexane extracts by UPLC-Q/TOF MS.

No.	T (min)	[M − H]− (*m*/*z*)	Molecular Formula	Theoretical Molecular	Relative Error (ppm)	MSn (*m*/*z*)	Compounds	Classification
				Weight				
1	8.55	251.1372	C_14_H_20_O_4_	251.1289	33	MS2: 207.1435; 153.0010	Tetradeca-trienedioic acid	Fatty acids
2	8.66	251.1372	C_14_H_20_O_4_	251.1289	33	MS2: 207.1435; 153.0010	Tetradeca-trienedioic acid isomer	Fatty acids
3	10.18	251.1372	C_14_H_20_O_4_	251.1289	33	MS2: 207.1435; 153.0010	Tetradeca-trienedioic acid isomer	Fatty acids
4	10.39	325.1252	C_19_H_18_O_5_	326.1154	30	MS2: 325.1252, 310.0999, 309.0922, 295.0750, 282.1047, 267.0828, 239.0883, 177.0358, 150.0489, 133.0463	Eucalyptol	Monoterpene
5	10.52	265.1544	C_15_H_22_O_4_	266.1517	10	-	Leptospermone	Sesquiterpenketone
6	10.74	489.3018	C_28_H_42_O_7_	490.293	18	MS2: 461.2899, 207.0286, 250.0839	Macrocarpal J/I	Sesquiterpene
7	12.49	485.2709	C_28_H_38_O_7_	486.2617	19	MS2: 207.0345, 183.0167	Eucalyptone Isomer	Phloroglucinol sesquiterpenoids
8	12.75	485.2709	C_28_H_38_O_7_	486.2617	19	MS2: 453.3354, 439.2457, 251.0918, 250.0839, 207.028	Eucalyptone	Phloroglucinol sesquiterpenoids
9	13.29	485.2709	C_28_H_38_O_7_	486.2617	19	MS1: 403.2620, 325.1956; MS2: 207.0345, 183.0167	Eucalyptone Isomer	Phloroglucinol sesquiterpenoids
10	13.46	455.3518	C_30_H_46_O_3_	456.3603	19	MS2: 455.3518, 407.3403, 363.3391, 248.9815	Ursolic/Oleanolic/Betulinic Acid	Triterpene
11	14.07	471.2937	C_28_H_40_O_6_	472.2825	24	MS1: 453.2439, 401.2091, 339.2095; MS2: 469.2737, 453.2439, 443.2924, 249.0845, 207.0374	Macrocarpal A/B/D/E	Phloroglucinol sesquiterpenoids
12	14.36	471.2937	C_28_H_40_O_6_	472.2825	24	MS2: 469.2737, 443.2924, 249.0845, 207.0374	Macrocarpal A/B/D/E Isomer	Phloroglucinol sesquiterpenoids
13	15.14	499.1778	C_26_H_28_O_10_	500.1682	19	MS1: 471.2937, 249.0845; MS2: 471.2937, 453.2814, 249.0845	Sideroxylonal A/B/C	Phloroglucinol sesquiterpenoids
14	15.46	471.2937	C_28_H_40_O_6_	472.2825	24	MS1: 339.2095; MS2: 207.0374	Macrocarpal A/B/D/E Isomer	Phloroglucinol sesquiterpenoids
15	15.85	487.343	C_30_H_48_O_5_	488.3501	15	-	Arjunolic/Asiatic Acid	Triterpene
16	16.31	471.2937	C_28_H_40_O_6_	472.2825	24	MS2: 469.2737, 443.2924, 249.0845, 207.0374	Macrocarpal A/B/D/E Isomer	Phloroglucinol sesquiterpenoids
17	16.61	471.2937	C_28_H_40_O_6_	472.2825	24	MS2: 469.2737, 453.3529, 249.0845, 207.0374	Macrocarpal A/B/D/E Isomer	Phloroglucinol sesquiterpenoids
18	16.82	471.2937	C_28_H_40_O_6_	472.2825	24	MS2: 469.2737, 453.3529, 249.0845, 207.0374	Macrocarpal A/B/D/E Isomer	Phloroglucinol sesquiterpenoids
19	17.65	471.2892	C_28_H_40_O_6_	472.2825	14	MS1: 401.2091, 385.2156, 325.0000; MS2: 469.2737, 249.0845, 207.0345	Macrocarpal A/B/D/E Isomer	Phloroglucinol sesquiterpenoids
20	18.12	471.347	C_30_H_48_O_4_	472.3552	17	MS2: 469.2737, 249.0845, 207.0374	Hydroxyursolic acid/Hederagenin	Triterpene
21	18.33	385.2156	C_23_H_30_O_5_	386.2093	16	-	Euglobal	Phloroglucinol sesquiterpenoids
22	18.56	497.3773	C_32_H_50_O_4_	498.3709	13	-	Oleanolic acid 3-acetate	Triterpene
23	19.16	467.2581	C_28_H_36_O_6_	468.2511	15	MS2: 471.2892, 207.0374, 249.9943	Unknown	Phloroglucinol sesquiterpenoids
24	19.8	469.2737	C_28_H_36_O_6_	470.2668	15	MS2: 325.0000, 265.1544 (423.0046)	Withanolide A/Eucalrobusone O	Phloroglucinol sesquiterpenoids
25	20.24	385.2156	C_23_H_30_O_5_	386.2093	16	-	Euglobal Isomer	Phloroglucinol sesquiterpenoids
26	20.45	453.3011	C_29_H_42_O_4_	454.3083	16	MS1: 385.2156, 311.1812, 249.9896	Unknown	-
27	20.7	453.2788	C_28_H_38_O_5_	454.2719	15	MS2: 207.0374, 250.0936	Macrocarpal C	Phloroglucinol sesquiterpenoids
28	21.06	453.2788	C_28_H_38_O_5_	454.2719	15	MS2: 207.0374, 250.0936	Macrocarpal C Isomer	Phloroglucinol sesquiterpenoids
29	21.28	453.2788	C_28_H_38_O_5_	454.2719	15	MS2: 207.0374, 250.0936	Macrocarpal C Isomer	Phloroglucinol sesquiterpenoids
30	21.43	453.2788	C_28_H_38_O_5_	454.2719	15	MS2: 207.0374, 250.0936	Macrocarpal C Isomer	Phloroglucinol sesquiterpenoids
31	21.74	453.2788	C_28_H_38_O_5_	454.2719	15	MS2: 207.0374, 250.0936	Macrocarpal C Isomer	Phloroglucinol sesquiterpenoids
32	21.98	453.2788	C_28_H_38_O=_5_	454.2719	15	MS2: 207.0374, 250.0936	Macrocarpal C Isomer	Phloroglucinol sesquiterpenoids
33	22.19	453.2788	C_28_H_38_O_5_	454.2719	15	MS2: 207.0374, 250.0936	Macrocarpal C Isomer	Phloroglucinol sesquiterpenoids
34	22.32	453.2788	C_28_H_38_O_5_	454.2719	15	MS2: 207.0374, 250.0936	Macrocarpal C Isomer	Phloroglucinol sesquiterpenoids
35	22.81	453.2788	C_28_H_38_O_5_	454.2719	15	MS2: 207.0374, 250.0936	Macrocarpal C Isomer	Phloroglucinol sesquiterpenoids
36	23.03	453.2788	C_28_H_38_O_5_	454.2719	15	MS2: 207.0374, 250.0936	Macrocarpal C Isomer	Phloroglucinol sesquiterpenoids
37	23.62	633.3807	C_39_H_54_O_7_	634.3869	10	MS1: 568.3217, 525.3054, 485.2456, MS2: 471.2018, 248.9832	3-O-trans-p-Coumaroyltormentic acid	Triterpene
38	24	703.3736	C_41_H_52_O_10_	704.356	25	MS2: 453.2788, 249.0845	Unknown	Diterpene Phenol
39	24.13	689.6077				MS2: 393.326, 207.0345	Unknown	Diterpene Phenol Sesquiterpene
40	25.23	775.5295	C_51_H_66_O_10_			-	Unknown	Dimethylated Diterpene Olefin
41	26.4	599.3742	C_39_H_52_O_5_	600.3814	12	-	Garcinialiptone	Triterpene
42	27.21	281.2483	C_18_H_34_O_2_	282.2558	27	MS2: 249.2125, 181.2023	Oleic Acid	Fatty Acid
43	28.27	485.2536	C_28_H_38_O_7_	486.2617	17	MS2: 325.1920, 207.0374	Eucalyptone Isomer	Formyl Sesquiterpene Phenol
44	28.92	469.2584	C_28_H_38_O_6_	470.2668	18	MS2: 423.2351	Withanolide A/Eucalrobusone O Isomer	Formyl Phloroglucinol Meroterpenoids
45	29	741.5692	C_50_H_32_O_10_			-	Unknown	Phloroglucinol sesquiterpenoids

Note: Relative error (ppm) = |actual value − theoretical value|/theoretical value × 10^6^.

**Table 2:** Compounds identified from *Eucalyptus* leaf 70% ethanol extracts by UPLC-Q/TOF MS.

No.	RT (min)	[M − H]^−^ (*m*/*z*)	Molecular Formula	Theoretical Molecular Weight	Relative Error (ppm)	MS^n^ (*m*/*z*)	Compound	Classification
1	0.51	353.1057	C_16_H_18_O_9_	354.0950	30	MS2: 165.0710, 203.0526, 233.0618, 259.0286, 275.1095, 335.0879	Chlorogenic Acid	Polyphenol
2	0.66	247.1153	C_14_H_16_O_4_	248.1048	42	MS2: 147.0990, 159.1346	Isohistidine	Amino Acid
3	0.98	497.1765	C_23_H_30_O_12_	498.1737	6	-	Eucaglobulin	Monoterpene
4	1.43	629.2004	C_31_H_34_O_14_	630.1948	9	MS2: 301.0416, 300.0337, 463.0988	Eucalmaidin D/Cypellogin A/B	Flavonoid
5	2.78	561.2329	C_26_H_42_O_13_	562.2625	53	MS2: 285.0383, 257.0797, 183.1026	19-Hydroxycinnzeylanol 19-Glucoside	Diterpene Glycoside
6	3.79	301.0025	C_14_H_6_O_8_	302.0062	12	275.0965, 273.0799	Ellagic acid	Polyphenol
7	7.80	487.3430	C_30_H_48_O_5_	488.3501	15	-	Arjunolic/Asiatic Acid	Triterpene
8	9.11	311.0909	C_18_H_16_O_5_	312.0997	28	-	Sideroxylin	Flavonoid
9	10.79	489.2883	C_28_H_42_O_7_	490.2930	10	MS2: 461.2899, 207.0286, 250.0839	Macrocarpal J/I	Phloroglucinol sesquiterpenoids
10	10.95	489.2883	C_28_H_42_O_7_	490.2930	10	MS2: 461.2899, 207.0286, 250.0839	Macrocarpal J/I Isomer	Phloroglucinol sesquiterpenoids
11	11.13	471.3470	C_30_H_48_O_4_	472.3552	17	MS2: 453.3354, 249.0748, 207.0286	Hydroxyursolic acid/Hederagenin	Triterpene
12	12.12	489.2838	C_28_H_42_O_7_	490.2930	19	MS2: 461.2899, 457.2581, 443.2794, 250.0839, 207.0286	Macrocarpal J/I Isomer	Phloroglucinol sesquiterpenoids
13	12.37	471.3470	C_30_H_48_O_4_	472.3552	17	MS2: 453.3354, 249.0748, 207.0286	Hydroxyursolic acid/Hederagenin Isomer	Triterpene
14	13.34	489.2883	C_28_H_42_O_7_	490.2930	10	MS2: 461.2899, 207.0286, 250.0839	Macrocarpal J/I Isomer	Phloroglucinol sesquiterpenoids
15	14.53	485.2528	C_28_H_38_O_7_	486.2617	18	MS2: 453.3354, 439.2457, 251.0918, 250.0839, 207.028	Eucalyptone	Phloroglucinol sesquiterpenoids
16	14.75	485.2528	C_28_H_38_O_7_	486.2617	18	MS2: 453.3354, 439.2457, 251.0918, 250.0839, 207.028	Eucalyptone Isomer	Phloroglucinol sesquiterpenoids
17	15.71	617.3841	C_39_H_54_O_6_	618.3920	13	MS1: 499.1595, 485.2528, 471.2759, 455.2471, 325.1800, 161.9348; MS2: 497.3271, 451.2456	O-p coumaroyl maslinic/alphitolic acid	Triterpene
18	16.12	471.3470	C_30_H_48_O_4_	472.3552	17	MS2: 453.3354, 249.0748, 207.0286	Hydroxyursolic acid/Hederagenin Isomer	Triterpene
19	16.24	471.3470	C_30_H_48_O_4_	472.3552	17	MS2: 453.3354, 249.0748, 207.0286	Hydroxyursolic acid/Hederagenin Isomer	Triterpene
20	16.39	471.3470	C_30_H_48_O_4_	472.3552	17	MS2: 453.3354, 249.0748, 207.0286	Hydroxyursolic acid/Hederagenin Isomer	Triterpene
21	16.87	471.2759	C_28_H_40_O_6_	472.2825	14	MS2: 469.2737, 443.2924, 249.0845, 207.0374	Macrocarpal A/B/D/E	Phloroglucinol sesquiterpenoids
22	17.07	455.3514	C_30_H_48_O_3_	456.3603	20	MS1: 369.8584, 339.1982, 311.1667	Ursolic/Oleanolic/Betulinic Acid	Triterpene
23	17.31	499.1778	C_26_H_28_O_10_	500.1682	19	MS1: 471.2937, 249.0845; MS2: 471.2937, 453.2814, 249.0845	Sideroxylonal A/B/C	Phloroglucinol sesquiterpenoids
24	17.68	471.2937	C_28_H_40_O_6_	472.2825	24	MS1: 453.2439, 401.2091, 339.2095; MS2: 469.2737, 453.2439, 443.2924, 249.0845, 207.0374	Macrocarpal A/B/D/E Isomer	Phloroglucinol sesquiterpenoids
25	17.86	471.2937	C_28_H_40_O_6_	472.2825	24	MS1: 453.2439, 401.2091, 339.2095; MS2: 469.2737, 453.2439, 443.2924, 249.0845, 207.0374	Isomer of Eucalyptal A/B/D/E	Phloroglucinol sesquiterpenoids
26	18.07	471.2937	C_28_H_40_O_6_	472.2825	24	MS1: 453.2439, 401.2091, 339.2095; MS2: 469.2737, 453.2439, 443.2924, 249.0845, 207.0374	Macrocarpal A/B/D/E Isomer	Phloroglucinol sesquiterpenoids
27	18.6	453.3354	C_30_H_46_O_3_	454.3446	20	MS1: 325.1846	Dehydroxyursolic Lactone	Triterpene
28	18.94	471.2937	C_28_H_40_O_6_	472.2825	24	MS1: 453.2439, 401.2091, 339.2095; MS2: 469.2737, 453.2439, 443.2924, 249.0845, 207.0374	Macrocarpal A/B/D/E Isomer	Phloroglucinol sesquiterpenoids
29	19.42	471.2937	C_28_H_40_O_6_	472.2825	24	MS1: 453.2439, 401.2091, 339.2095; MS2: 469.2737, 453.2439, 443.2924, 249.0845, 207.0374	Macrocarpal A/B/D/E Isomer	Phloroglucinol sesquiterpenoids
30	20.12	499.1778	C_26_H_28_O_10_	500.1682	19	MS1: 471.2937, 249.0845; MS2: 471.2937, 453.2814, 249.0845	Sideroxylonal A/B/C Isomer	Phloroglucinol sesquiterpenoids
31	20.28	469.2560	C_28_H_38_O_6_	470.2668	23	MS1: 443.0080, 325.1809, 265.1477; MS2: 425.2687	Withanolide A/Eucalrobusone O	Formyl Phloroglucinol Meroterpenoids
32	20.4	599.3732	C_39_H_52_O_5_	600.3814	14	MS1: 455.2475	Garcinialiptone	Triterpene
33	20.78	629.2004	C_31_H_34_O_14_	630.1948	9	MS2: 301.0416, 300.0337, 463.0988	Eucalmaidin D/Cypellogin A/B	Flavonoid
34	21.06	453.2788	C_28_H_38_O_5_	454.2719	15	MS2: 207.0374, 250.0936	Macrocarpal C	Phloroglucinol sesquiterpenoids
35	22.57	453.2788	C_28_H_38_O_5_	454.2719	15	MS2: 207.0374, 250.0936	Macrocarpal C Isomer	Phloroglucinol sesquiterpenoids
36	24.11	453.2788	C_28_H_38_O_5_	454.2719	15	MS2: 207.0374, 250.0936	Macrocarpal C Isomer	Phloroglucinol sesquiterpenoids
37	24.6	453.2788	C_28_H_38_O_5_	454.2719	15	MS2: 207.0374, 250.0936	Macrocarpal C Isomer	Phloroglucinol sesquiterpenoids
38	25.16	453.2788	C_28_H_38_O_5_	454.2719	15	MS2: 207.0374, 250.0936	Macrocarpal C Isomer	Phloroglucinol sesquiterpenoids
39	25.42	453.2788	C_28_H_38_O_5_	454.2719	15	MS2: 207.0374, 250.0936	Macrocarpal C Isomer	Phloroglucinol sesquiterpenoids
40	25.83	453.2788	C_28_H_38_O_5_	454.2719	15	MS2: 207.0374, 250.0936	Macrocarpal C Isomer	Phloroglucinol sesquiterpenoids
41	26.69	607.3989	C_38_H_56_O_6_	608.4077	14	MS2: 249.0845, 207.0374	Sesquiterpene Alcohol Ester	Sesquiterpene Phenol Alcohol
42	26.95	453.2657	C_28_H_38_O_5_	454.2719	14	MS2: 207.0374, 250.0936	Macrocarpal C Isomer	Phloroglucinol sesquiterpenoids

Note: relative error (ppm) = |actual value − theoretical value|/theoretical value × 10^6^.

**Table 3:** Summary of the proportions of components in eucalyptus leaves.

Composition	Content/%	Summation/%
Crude Fiber	27.60	76.99
Crude Protein	5.64	
Crude Fat	4.85	
Crude Ash	4.10	
Moisture	18.40	
Total Sugars	6.86	
Total Polyphenols	4.77	
Ursolic Acid	0.586	
Total Terpenes	2.84	
Phloroglucinols	1.93	

**Table 4:** Inhibitory effect of the triterpenoids and positive control drug against human cancer cell MDA-MB-231, SGC-7901 and Hela.

Compound	Concentration /μg∙mL^−1^	Inhibition Ratio/%			IC_50_/μg∙mL^−1^		
		MDA-MB- 231	SGC- 7901	Hela	MDA-MB- 231	SGC- 7901	Hela
Triterpenoids	0.00	0.00	0.00	0.00	50.67	43.12	42.65
	6.25	16.15	4.62	5.17			
	12.50	32.01	4.24	9.83			
	25.00	29.52	28.90	16.73			
	50.00	46.63	62.87	57.97			
	100.00	64.01	68.96	77.13			
5-FU	0.00	0.00	0.00	0.00	2.36	12.61	22.81
	6.25	60.95	35.05	17.47			
	12.50	69.71	35.99	22.89			
	25.00	73.26	38.72	34.07			
	50.00	73.12	41.48	36.25			
	100.00	73.04	50.26	40.07			

## Data Availability

Data are contained within the article.
